# Hyperchaotic System-Based PRNG and S-Box Design for a Novel Secure Image Encryption

**DOI:** 10.3390/e27030299

**Published:** 2025-03-13

**Authors:** Erman Özpolat, Vedat Çelik, Arif Gülten

**Affiliations:** 1 Department of Electrical-Electronics Engineering, Faculty of Engineering and Architecture, Mus Alparslan University, Mus 49100, Turkey; 2 Department of Electrical-Electronics Engineering, Faculty of Engineering, Firat University, Elazig 23119, Turkey; celik@firat.edu.tr (V.Ç.); agulten@firat.edu.tr (A.G.)

**Keywords:** hyperchaotic systems, pseudo-random number generator (PRNG), S-box, image encryption, chaotic cryptography

## Abstract

A hyperchaotic system was analyzed in this study, and its hyperchaotic behavior was confirmed through dynamic analysis. The system was utilized to develop a pseudo-random number generator (PRNG), whose statistical reliability was validated through NIST SP800-22 tests, demonstrating its suitability for cryptographic applications. Additionally, a 16 × 16 S-box was constructed based on the hyperchaotic system, ensuring high nonlinearity and strong cryptographic performance. A comparative analysis revealed that the proposed S-box structure outperforms existing designs in terms of security and efficiency. A new image encryption algorithm was designed using the PRNG and S-box, and its performance was evaluated on 512 × 512 grayscale images, including the commonly used baboon and pepper images. The decryption process successfully restored the original images, confirming the encryption scheme’s reliability. Security evaluations, including histogram analysis, entropy measurement, correlation analysis, and resistance to differential and noise attacks, were conducted. The findings showed that the suggested encryption algorithm outperforms current techniques in terms of security and efficiency. This study contributes to the advancement of robust PRNG generation, secure S-box design, and efficient image encryption algorithms using hyperchaotic systems, offering a promising approach for secure communication and data protection.

## 1. Introduction

In recent years, the capacity of digital data has increased significantly, and with the advancement of electronic devices, large volumes of data are continuously transmitted [1]. This situation has created various security risks, particularly concerning image and video data. Traditional encryption methods such as DES and AES have demonstrated strong performance when encoding text; however, they are not the optimal choice for encrypting large-scale contemporary image data [2,3]. Consequently, research efforts have been initiated to develop alternative methods for image encryption, one of which is chaos-based encryption.

Recent years have seen a notable increase in interest in the use of chaotic systems in cryptography applications [4,5,6]. Because it offers robust key generation mechanisms, chaos—a complex process that seems random in deterministic nonlinear systems—is very beneficial for encryption systems. Chaos is prevalent in various natural and societal processes, which has led to its widespread application and attracted researchers across multiple disciplines [7]. The fundamental requirements of cryptographic systems are well served by the characteristics of chaotic systems, such as randomness, ergodicity, sensitivity to control parameters, and dependency on initial conditions. Encryption techniques benefit greatly from the deterministic but extremely unpredictable values produced by chaotic systems [8,9]. Applications for encryption that take advantage of these chaotic features have been created [10,11,12]. Furthermore, image encryption methods based on low-dimensional chaotic maps are less safe than those based on high-dimensional chaotic systems. Recent studies have demonstrated innovative approaches in the field of image encryption, contributing to enhancing the security and performance of cryptographic systems [13,14]. For security-critical applications like image encryption, high-dimensional chaotic systems—especially hyperchaotic ones—offer significant benefits because of their broad key space, high sensitivity, intricate dynamic behavior, and increased randomness. High-dimensional chaotic systems are difficult to decrypt using general techniques like phase space reconstruction and nonlinear prediction, which are effective in decrypting low-dimensional chaotic maps [15]. A hyperchaotic system is defined mathematically as a chaotic system with multiple positive Lyapunov exponents, which indicates that its dynamics simultaneously change in multiple directions. Consequently, compared to a typical chaotic system, a hyperchaotic attractor displays a more complex dynamic behavior [16,17,18]. This expansion of dynamic behavior in multiple directions simultaneously makes hyperchaotic systems superior to chaotic systems in various chaos-based applications, including technological implementations. For instance, hyperchaotic systems can be utilized in communication systems to enhance information security due to their higher unpredictability and more complex attractor structures. Messages encrypted with chaotic systems are not always secure in encryption approaches that use the chaotic attractor to encode the transmitted message. Consequently, compared to chaotic systems, hyperchaotic systems provide a more detailed topological structure and a more complex dynamic behavior. Many scientific and engineering sectors are now interested in hyperchaos, which is why its use in chaos-based cryptography is becoming more and more common [19]. In contrast to hyperchaotic systems, traditional chaotic attractors have a well-known drawback in topological applications: they only have one positive Lyapunov exponent (LE), which results in a reduced degree of randomness [20,21].

By leveraging the fundamental properties of chaotic systems, robust pseudo-random number generators (PRNGs) and highly secure S-box structures can be designed. PRNGs play a crucial role in generating random keys, while S-box components enhance encryption algorithm security by providing nonlinear transformations. Consequently, the application of hyperchaotic systems in these areas presents significant advantages over that of existing encryption techniques. Encryption schemes based on hyper chaotic system state variables generate random number sequences for cryptographic applications [22,23]. The greater the encryption complexity, the more random the generated numbers become. One typical application of chaotic systems in encryption involves the development of chaos-based random number generators [24,25,26,27,28,29]. In a study by Tuna [30], artificial neural network (ANN)-based 2D chaotic oscillators and ring oscillator structures were used to propose a novel real-time, fast, and robust chaos-based PRNG. By extracting several bits in every iteration from the fractional portion of a chaotic map, Moysis et al. [31] presented a straightforward technique for creating a pseudo-random bit generator. Shi et al. [32] suggested a new PRNG that combines the three-dimensional variables of a cat chaotic map. A review of recent studies indicates that chaotic systems are commonly used to generate pseudo-random number sequences.

By hiding the connection between the plaintext and the cipher text, a substitution box (S-box), a nonlinear element used in block ciphers, significantly improves cryptographic security. In sophisticated cryptosystems, S-boxes built with various algebraic structures are regarded as one of the most dependable encryption elements. Due to their significance and practicality in cryptographic systems, numerous researchers have employed S-boxes in various image encryption schemes [7]. Liu et al. [33] created a three-dimensional improved quadratic map (3D-IQM)-based cryptographically robust S-box. In his study, Khan [34] proposed a chaotic-based S-box design aimed at simplifying the encryption process while enhancing security and reducing computational complexity. Wang and Wang [35] presented an approach for encrypting images that uses dynamic S-boxes produced by chaotic systems. These researchers successfully managed to determine the parameters and beginning states of chaotic systems for the first S-box by using the last pixel of the plaintext image and an external 256-bit key. Islam and Liu [36] efficiently generated a set of cryptographically strong S-boxes using a recently discovered four-dimensional hyperchaotic system. Hyperchaotic systems are still neglected in these techniques, whereas chaotic systems have been widely employed. Furthermore, some S-boxes created using the current techniques do not perform well in cryptography.

Furthermore, recent research highlights the growing importance of image encryption applications employing chaos-based PRNGs and S-boxes. Vijayakumar and Ahilan [37] proposed a novel encryption technology based on chaotic map substitution boxes (S-boxes) and cellular automata (CA) to overcome challenges commonly encountered in chaotic encryption schemes. To address the inadequate randomness provided by one-dimensional chaotic maps and the vulnerabilities of software-based approaches, they introduced a four-dimensional memristive hyperchaotic system, offering a superior chaotic range, increased unpredictability, and enhanced ergodicity. In their study, Wu and Kong [38] proposed a new 2D hyperchaotic map with holistic advantages over conventional two-dimensional hyperchaotic maps. They used this hyperchaotic system to generate an S-box and develop an image encryption algorithm. Singh et al. [39] presented an encryption technique for images based on dynamically generated substitution boxes (S-boxes) and elliptic curve points over a finite field. Yang et al. [40] suggested a four-dimensional hyperchaotic system-based S-box creation algorithm and enhanced particle swarm optimization, utilizing their created S-box for image encryption. Yang et al. [41] constructed a novel two-dimensional discrete hyperchaotic map with a linearly cross-linked topological structure combining tent and logistic maps. They then generated a PRNG based on their proposed hyperchaotic map and applied the generated random numbers to image encryption.

**Figure 1:**
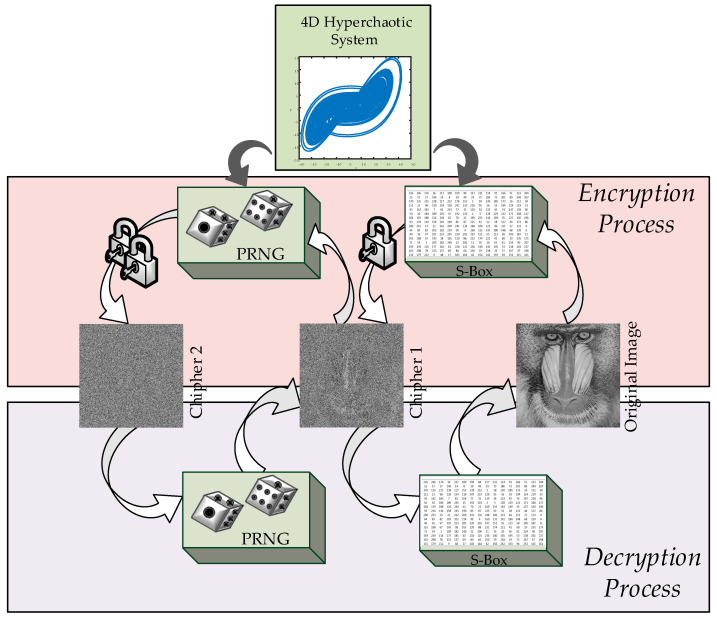
Visual representation of the research.

The main motivation of the authors is the existence of few studies in the literature on PRNG and S-box design of hyperchaotic systems. Therefore, in this study, the dynamic properties of a hyperchaotic system previously introduced by the authors were analyzed [42]. First, based on the state variables of the hyperchaotic system, a PRNG was created, and NIST tests were carried out. Additionally, an S-box was constructed using the hyperchaotic system, and its performance was evaluated. The developed PRNG and S-box were then used to propose a novel encryption algorithm, which was applied to an image encryption scheme. Furthermore, encryption tests were conducted, yielding successful results. Given the high-security advantages offered by hyperchaotic systems, this study is expected to provide a significant contribution to chaos-based encryption methods. The proposed encryption scheme also aims to provide a theoretical framework for the implementation of S-box design, pseudo-random number generation, and image encryption on hardware-based platforms such as FPGAs. A graphical representation of the study is presented in Figure 1.

This paper’s remaining sections are organized as follows: The mathematical model and dynamic analysis of the hyperchaotic system are shown in Section 2. The PRNG’s design and assessment, including the outcomes of the NIST SP800-22 test, are covered in Section 3. The S-box design and performance analysis are explained in Section 4. The recently created image encryption and decryption algorithm is presented in Section 5. The encryption performance testing and simulation results are presented in Section 6. Lastly, the conclusions are presented in Section 7.

## 2. The Hyperchaotic System

The hyperchaotic system used in this study was previously introduced by the authors as a novel contribution to the literature in another research work [42]. The mathematical expressions defining the hyperchaotic system are presented in (1)

(1) $$\begin{array}{c} \dot{x}=-24x+8y\\ \dot{y}=ax+y-2xz\\ \dot{z}=bxy-4z+w\\ \dot{w}=-xy-2z-w \end{array}$$

In this system, the parameters a and b are positive constants, while x, y, z, and w represent the state variables of the hyperchaotic system. Under specific values of a and b and appropriate initial conditions, the system exhibits hyperchaotic behavior. A key characteristic of this system is its high sensitivity to the initial conditions, resulting in significantly different system behaviors for varying initial values.

### Dynamic Analysis of the Hyperchaotic System

In this section, several dynamic analyses of the hyperchaotic system are presented. Dynamic analyses are essential for determining whether a system exhibits chaotic behavior and for identifying its hyperchaotic properties.

One of the most critical dynamic analyses for hyperchaotic systems is the bifurcation diagram analysis. Bifurcation diagrams help examine the chaotic behavior of a system by analyzing how its dynamics change with respect to variations in parameter values. They also aid in determining the appropriate parameter ranges for the system.

**Figure 2:**
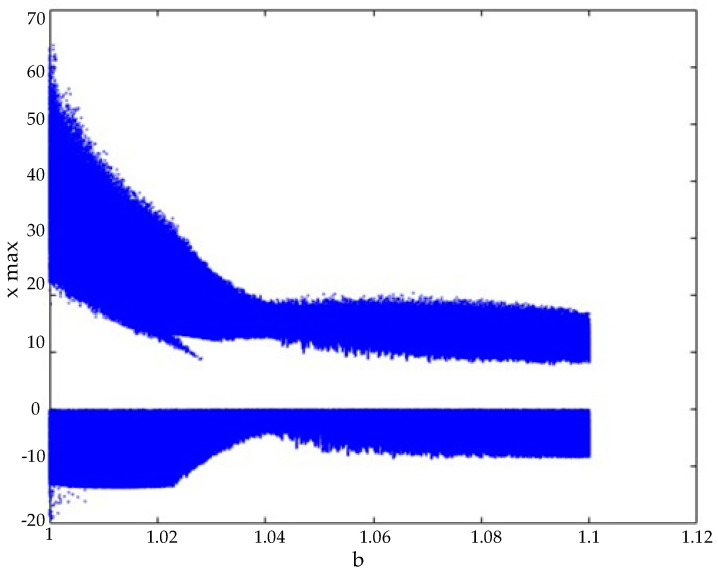
Bifurcation diagram.

Regarding the hyperchaotic system’s bifurcation diagram, the parameter *a* was fixed at 20, while the parameter *b* was varied between 1 and 1.1. The system’s initial conditions were set as $\left[x_{0}\;y_{0}\;z_{0}\;w_{0}\right]=[2\;2\;2\;2]$, and calculations were performed accordingly. The resulting bifurcation diagram is illustrated in Figure 2.

Upon examining the bifurcation diagram, it was observed that the system exhibits chaotic behavior for values of *b* between 1 and 1.1. Based on this observation, the parameter was set to b=40/39. Throughout the remainder of this study, the hyperchaotic system parameters were fixed as a=20 and b=40/39.

Another crucial dynamic analysis for hyperchaotic systems is the Lyapunov exponent analysis. Lyapunov exponents are used to determine whether a system is chaotic or hyperchaotic. A system is considered hyperchaotic if it possesses two or more positive Lyapunov exponents.

**Figure 3:**
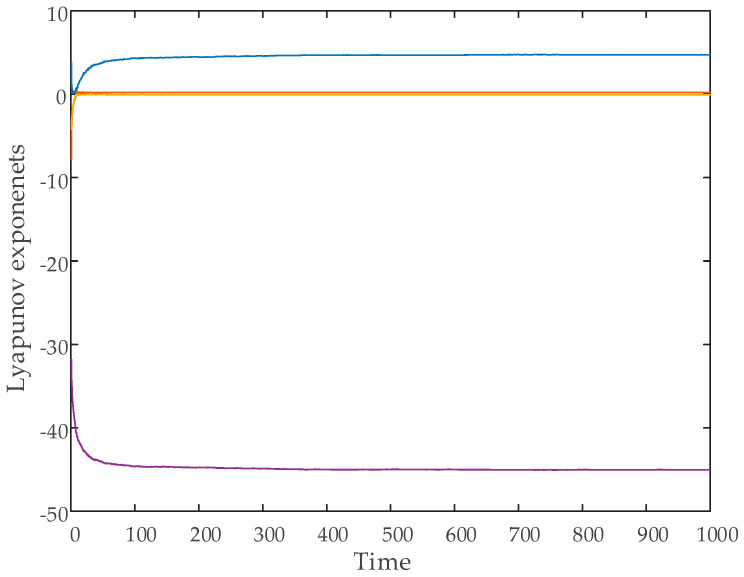
Time evolution of Lyapunov exponents.

In this study, the Wolf algorithm was employed to compute the Lyapunov exponents [43]. The variation of the Lyapunov exponents over time for the hyperchaotic system is presented in Figure 3. The calculations were performed using the initial conditions $\left[x_{0}\;y_{0}\;z_{0}\;w_{0}\right]=[2\;2\;2\;2]$.

The computed Lyapunov exponents for the system are as follows:

$$L_{1}=4.733,\;L_{2}=0.065,\;L_{3}=0,\;L_{4}=-45.040$$

The system was categorized as hyperchaotic because of its two positive Lyapunov exponents.

**Figure 4:**
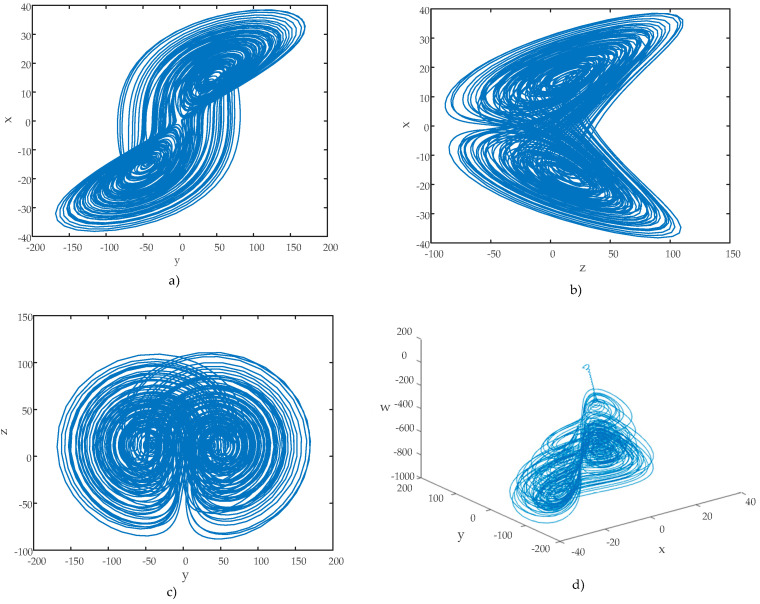
Phase–space diagrams. (**a**) y−x Phase diagram, (**b**) z−x phase diagram, (**c**) y−z phase diagram, (**d**) x−y−w 3D phase portrait.

Phase–space diagrams are another essential tool for analyzing the chaotic behavior of hyperchaotic systems. These diagrams provide significant insights into a system’s dynamics. The phase–space diagrams of the hyperchaotic system are shown in Figure 4. These diagrams were generated using the initial conditions $\left[x_{0}\;y_{0}\;z_{0}\;w_{0}\right]=[2\;2\;2\;2]$.

**Figure 5:**
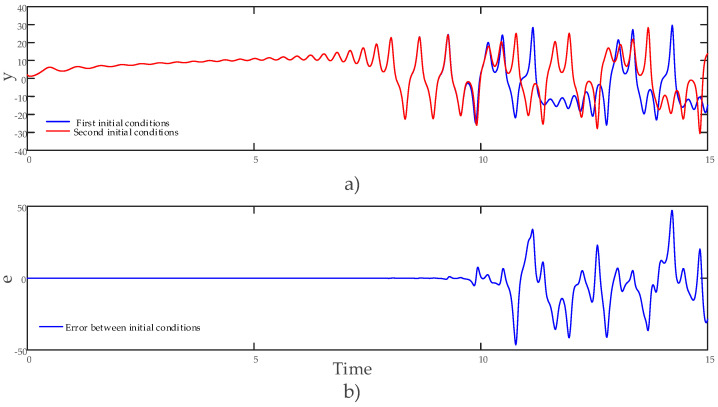
(**a**) The y state variable’s time series for closely spaced initial conditions and (**b**) the y state variable’s error variation for closely spaced initial conditions.

An examination of the phase diagrams revealed that the hyperchaotic system possesses strange attractors, which further confirmed its hyperchaotic nature. Furthermore, Figure 5 shows the time series of the state variable y and the change of the error when [2 2 2 2] and [2.01 2 2 2] are selected, so that the initial conditions are very close.

The conducted analyses demonstrated that the system exhibits hyperchaotic behavior. More extensive dynamic analyses of the hyperchaotic system can be found in the authors’ previous study [42].

## 3. Pseudo-Random Number Generation (PRNG)

At this stage of the study, pseudo-random number generation was performed using the state variables of the hyperchaotic system. Pseudo-random number sequences generated based on chaos play a crucial role in cryptography [44]. The numbers that are produced must have high statistical qualities, be surprising, and not be replicable. The state variables went through a preprocessing phase as outlined in (2) in order to produce pseudo-random numbers using the hyperchaotic system. The calculations were performed using the system parameters a=20 and b=40/39 and the initial conditions $\left[x_{0}\;y_{0}\;z_{0}\;w_{0}\right]=[1\;1\;1\;1]$.

(2) $$\begin{array}{c} R_{x}=[((x_{i}-floor(x_{i}))\times 10^{14}]\\ R_{y}=[((y_{i}-floor(y_{i}))\times 10^{14}]\\ R_{z}=[((z_{i}-floor(z_{i}))\times 10^{14}]\\ R_{w}=[((w_{i}-floor(w_{i}))\times 10^{14}] \end{array}$$

First, the decimal part of the state variables was extracted by subtracting the nearest integer value. These extracted values were then multiplied by $10^{14}$ to scale them up and converted into the integer form. Subsequently, the transformed state variable values were XORed among themselves to generate pseudo-random numbers. The structure of this transformation is presented in (3)

(3) $$P=mod(bitxor\left(bitxor\left(R_{x},R_{y}\right),bitxor\left(R_{z},R_{w}\right)\right),256)$$

To assess the randomness of the generated pseudo-random numbers, a number of statistical tests were employed [45]. In this study, the generated pseudo-random numbers were subjected to the NIST SP800-22 test suite, and the results are presented in Table 1. Since the NIST SP800-22 test requires at least one million bits of data, a dataset of over 1.5 million bits was generated and tested.

The generated pseudo-random numbers successfully passed all 15 tests conducted in the NIST SP800-22 test suite.

## 4. S-Box Design Based on the Hyperchaotic System and Performance Analysis

At this stage, an S-box was designed using the hyperchaotic system, followed by performance tests. One of the most critical components of block cipher techniques is the S-box, which introduces confusion into the encryption process. Consequently, utilizing a robust S-box structure contributes significantly to secure encryption [26].

During the S-box design process, the hyperchaotic system parameters were set as a=20 and b=40/39, and the initial conditions were chosen as $\left[x_{0}\;y_{0}\;z_{0}\;w_{0}\right]=[1\;1\;1\;1]$. The designed S-box was constructed as a 16×16 matrix containing non-repeating values ranging between 0 and 255. 

For the S-box construction, the values of the x state variable from the hyperchaotic system were utilized. The pseudocode of the S-box generation algorithm is presented in Algorithm 1.
**Algorithm 1.** The S-box generation algorithm pseudocode1: **Start**2: Define the hyperchaotic system equations3: Set initial conditions and parameters4: Use the ODE45 technique to solve the problem and obtain the time series x5: Resample the time steps for uniform distribution6: Discard the first 6000 iterations to eliminate transient effects7: Select the next 256 points from x for S-box construction8: Extract the first state variable (x values) from the resampled data9: Sort the first state variable x and obtain index values10: Rearrange the integer set [0–255] based on the sorted indices to generate the S-box11: Ensure that all values in the S-box are unique12: Reshape the S-box into a 16 × 16 matrix13: The 16 × 16 chaos-based S-box is ready to use with
x
14: **End**

Hyperchaotic systems require a certain amount of time to reach stability. Therefore, the first 6000 iterations of the x state variable were discarded, and a dataset consisting of the next 256 points was selected. Using this dataset, an index set was generated as k=sort(x).

Using these indices, the integer set D=0:255 was rearranged, and the S-box was constructed based on S=D(k). This approach ensured that all values within the S-box were unique.

Finally, the obtained S-box was formatted into a 16×16 matrix, making it ready for use. As a result, a hyperchaos-based, randomly distributed, and nonlinear S-box was successfully generated. The designed 16×16 S-box is presented in Table 2.

To provide robust encryption capabilities, S-box structures need to pass specific performance requirements. Nonlinearity, the bit independence criterion (BIC), the differential approximation probability (DP), the linear approximation probability (LP), and the rigorous avalanche criterion (SAC) are some of these tests. These tests must be passed by a strong S-box design.

Nonlinearity is one of the most important performance tests. A function’s resilience to linear and correlation attacks increases with its nonlinear property [46]. The nonlinearity of Boolean functions is measured using the Walsh spectrum. The highest nonlinearity value for symmetric Boolean functions is 112, while nonlinearity values higher than 98 are regarded as substantial [47].

For computational efficiency, the Walsh spectrum-based definition of nonlinearity is used. The nonlinearity of an n-bit Boolean function f(x) is defined by (4)

(4) $$N_{f}=2^{n-1}-\frac{1}{2}\underset{\omega \in F_{2}^{n}}{\max}\left|S_{\left(f\right)}(\omega )\right|\;$$

Here, $S_{\left(f\right)}(\omega )$ represents the Walsh spectral component of f(x). The value of $S_{\left(f\right)}(\omega )$ is computed using (5)

(5) $$S_{\left(f\right)}\left(w\right)=\sum_{x\in F_{2}^{n}}{\left(-1\right)}^{f\left(x\right)⊕x\cdot \omega }$$

Here, x⋅ω represents the dot product of the vectors ω and x, where $\omega \in F_{2}^{n}$.

For each Boolean function, eight nonlinearity values were calculated, along with their average value, which is presented in Table 3.

The minimum nonlinearity value was calculated as 102, the maximum as 108, and the average as 105.5. These results indicate that the proposed S-box exhibits a high degree of nonlinearity.

Another performance criterion is the strict avalanche criterion (SAC). Webster and Tavares introduced the SAC, which combines completeness and avalanche effect [48]. According to the SAC, the likelihood of each output bit flipping is 0.5, suggesting that when one input bit of a Boolean function is flipped, half of the output bits should change. An independence matrix was used to calculate the S-box SAC value. If an S-box closely satisfies the SAC, each element in the independence matrix should be close to 0.5. The independence matrix of the proposed S-box is presented in Table 4.

The average SAC value was calculated as 0.4980, which is very close to 0.5. This result confirmed that the proposed S-box satisfies the strict avalanche criterion (SAC).

Another cryptographic property introduced by Webster and Tavares is the bit independence criterion (BIC) [48]. For any two outputs of an S-box, represented as the Boolean functions $f_{i}\left(x\right)$ and $f_{j}\left(x\right)$ where (i≠j, i≥1, j≤n), the BIC of nonlinearity states that if an S-box satisfies the BIC, then the function $f_{i}\left(x\right)⊕f_{j}\left(x\right)$ should also exhibit nonlinearity.

Similarly, if an S-box satisfies the BIC–strict avalanche criterion (BIC-SAC), then $f_{i}\left(x\right)⊕f_{j}\left(x\right)$ must also satisfy the SAC property. The obtained results for these evaluations are presented in Table 5 and Table 6.

The BIC-NL matrix’s average value, as determined by looking at Table 5, was 103.29, while Table 6 shows that the average value of the BIC-SAC matrix was calculated as 0.4976.

For BIC-NL, the ideal value is above 100, and the obtained result met this requirement. Additionally, the BIC-SAC value was very close to the ideal value of 0.5. Based on these findings, it can be concluded that the proposed S-box exhibits strong BIC properties.

The differential probability (DP) criterion is used to assess an S-box’s differential resistance. The S-box will withstand differential attacks if the relationship between the input and the output bits is evenly distributed. The imbalance in the input/output XOR distribution table served as the basis for Biham and Shamir’s differential cryptanalysis proposal [49]. The DP value is calculated using the formula given in Equation (6).

(6) $$DP=\underset{∆x\neq 0,∆y}{\max}\left(\frac{⋕\left\{x\in N|S(x)⊕S(x⊕∆x)=∆y\right\}}{2^{n}}\right)$$

Here, $\Delta x=x⊕x^{\prime }$ and $\Delta y=y⊕x^{\prime }$ represent the differential values for the input pair $\left(x,\;x^{\prime }\right)$ and the output pair $\left(y,\;y^{\prime }\right)$, respectively. S(x) denotes the transformation of the input by the S-box.

Differential cryptanalysis is more difficult to perform on an S-box with a lower DP value. An input–output XOR distribution table with equal probabilities was computed, and the maximum value was considered for evaluating this criterion. Table 7 displays the DP distribution table for the planned S-box.

The maximum value in the input–output XOR distribution table of the S-box is 12. The maximum computed DP value for the proposed S-box is 0.0469.

A secure cryptosystem must exhibit strong diffusion and confusion properties. Robust S-boxes ensure that cryptosystems achieve strong diffusion effects and confusion by implementing a nonlinear mapping between input and output data. The nonlinear mapping property of an S-box and its resistance to linear cryptanalysis increase as the linear probability (LP) decreases [50]. The LP value is calculated using the formula given in (7)

(7) $$LP=\underset{\alpha_{x},\beta_{x}\neq 0}{\max}\left|\frac{⋕\left\{x\in N|x\cdot \alpha_{x}=S(x)\cdot \beta_{x}\right\}}{2^{n}}-\frac{1}{2}\right|$$

Here, N={0, 1, ... , 255} represents the input space, while $\alpha_{x}$ and $\beta_{x}$ are the input and output masks, respectively $\left(\alpha_{x}\in N,\;\beta_{x}\in N\right)$. The “∙” operator denotes the scalar product, and x∈N|x represents the count of values x satisfying condition X. The smaller the LP value, the higher the resistance against linear attacks. In this study, the maximum LP value of the designed S-box was found to be 0.1406. A comparative analysis of the S-box designed in this study with other S-box structures proposed in the literature is presented in Table 8.

According to the performance tests, the suggested S-box in this work outperforms several existing designs described in the literature in terms of encryption strength. This outcome suggests that the suggested S-box offers a strong basis for further investigation into encryption techniques.

## 5. An Innovative Image Encryption Method and Its Application Using the Dynamic S-Box and PRNGs

At this stage of the study, an image encryption and decryption application was implemented using the S-box and pseudo-random numbers generated from the previously hyperchaotic system, which successfully passed the performance tests. Subsequently, encryption performance tests were conducted.

**Figure 6:**
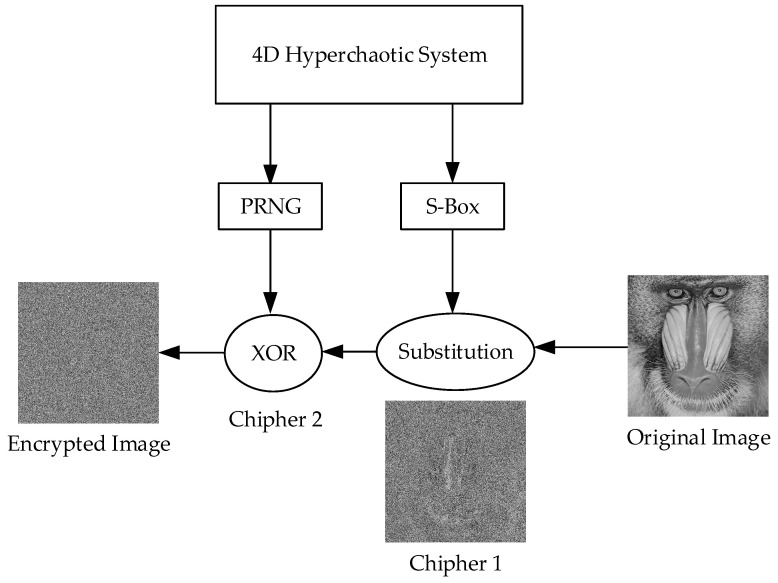
Encryption structure using S-box and PRNG.

The proposed encryption structure is illustrated in Figure 6.

Encryption Process Algorithm

Step 1: Loading and preprocessing the original image

In the first stage of the encryption process, a grayscale image to be used as input is loaded into the system. If the input image is in RGB (color) format, it is converted to grayscale, ensuring that the pixel values are processed through a single channel. This ensures that the encryption process only operates on luminance levels. The image is stored as a matrix for ease of processing.

Step 2: Generating randomness using the hyperchaotic system

The hyperchaotic system is used as the randomness source. The initial conditions of the system are set, and the differential equations are solved using numerical solvers such as ode45. All iterations of the hyperchaotic system are recorded, as they are used for both S-box generation and PRNG creation.

Step 3: Dynamic S-box generation

The x state variable obtained from the hyperchaotic system is used to construct the S-box. Since hyperchaotic systems are sensitive to the initial conditions, the first 6000 iterations are discarded to eliminate instability. The remaining values are sorted, and an index array is obtained. Using these indices, a random permutation within the 0-255 range is generated, creating a dynamic S-box. The S-box is formatted into a 16 × 16 matrix for a structured representation. The S-box generation process is described in detail in Section 4.

Step 4: Encrypting image pixels using the S-box

**Figure 7:**
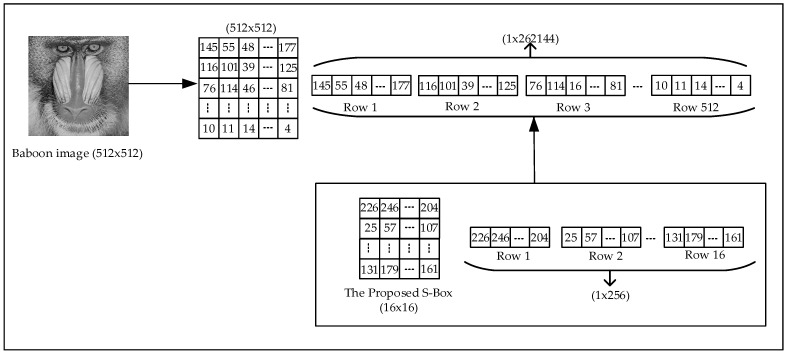
Encryption using the S-box structure.

The image is first converted into a one-dimensional vector, and each pixel value is replaced by its corresponding value in the S-box, performing the first encryption phase. This process follows the substitution (S-box transformation) principle, ensuring that the pixel values are rearranged in a completely different order. The encryption process is illustrated in Figure 7.

Step 5: Generating a random matrix using the hyperchaotic system

The decimal part of the x, y, z, w state variables from the hyperchaotic system was extracted to generate random numbers. The detailed process of random number generation was explained in Section 3. The extracted values undergo a bitwise XOR operation, forming a strong PRNG. The PRNG sequence is converted into a 512×512 random matrix, ensuring that it matches the dimensions of the encrypted image.

Step 6: Second layer of encryption using the XOR operation

To add an additional layer of security, the S-box-encrypted image is bitwise XORed with the generated random matrix. This process ensures that each pixel value undergoes an additional encryption step, resulting in the final encrypted image.

**Figure 8:**
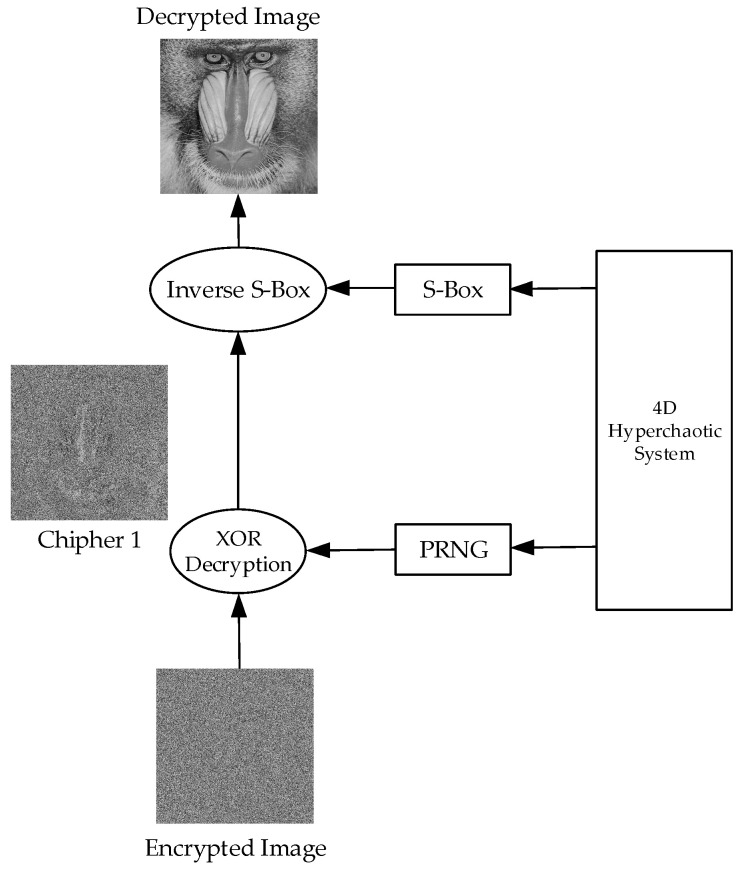
Method for extracting the original image from the encrypted image.

Figure 8 illustrates the structure for extracting the original image from the encrypted image.

The decryption process consists of reversing the encryption steps to reconstruct the original image. The first step is to regenerate the random matrix (PRNG) using the same hyperchaotic system and initial conditions as in encryption. The encrypted image is then subjected to a bitwise XOR operation with this random matrix, yielding the Cipher 1 image, which was only encrypted using the S-box transformation. Next, the inverse S-box transformation is applied, mapping each pixel value back to its original state. Finally, all pixels are restored to their original positions, resulting in a fully reconstructed original image. This process ensures that the decryption is lossless, provided that all steps are executed correctly and in sync.

## 6. Simulation Results and Encryption Performance Tests

At this stage of the study, the proposed encryption and decryption algorithms were tested, and performance evaluations related to the encryption process were conducted. The MATLAB 2021a platform was used for the simulations. The hyperchaotic system parameters were set to a=20, b=40/39, with initial conditions $\left[x_{0}\;y_{0}\;z_{0}\;w_{0}\right]=[1\;1\;1\;1]$.

### 6.1. Encryption and Decryption Simulations

**Figure 9:**
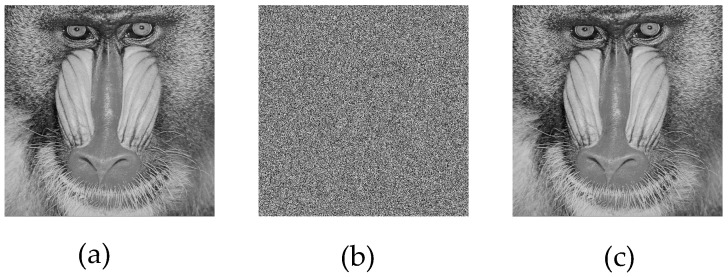
(**a**) Original baboon image, (**b**) encrypted baboon image, (**c**) decrypted baboon image.

**Figure 10:**
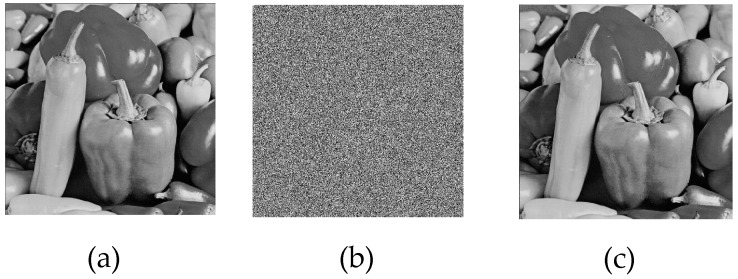
(**a**) Original pepper image, (**b**) encrypted pepper image, (**c**) decrypted pepper image.

For the simulation, 512 × 512 grayscale baboon and pepper images, commonly used in image encryption research, were selected. The results obtained from the application are presented in Figure 9 and Figure 10.

Upon analyzing the results, the 512 × 512 grayscale baboon and pepper images were successfully encrypted and subsequently decrypted. While visual inspection suggested that the encryption was performed correctly, additional encryption performance tests were required to quantify the effectiveness of the proposed method.

### 6.2. Histogram Analysis

**Figure 11:**
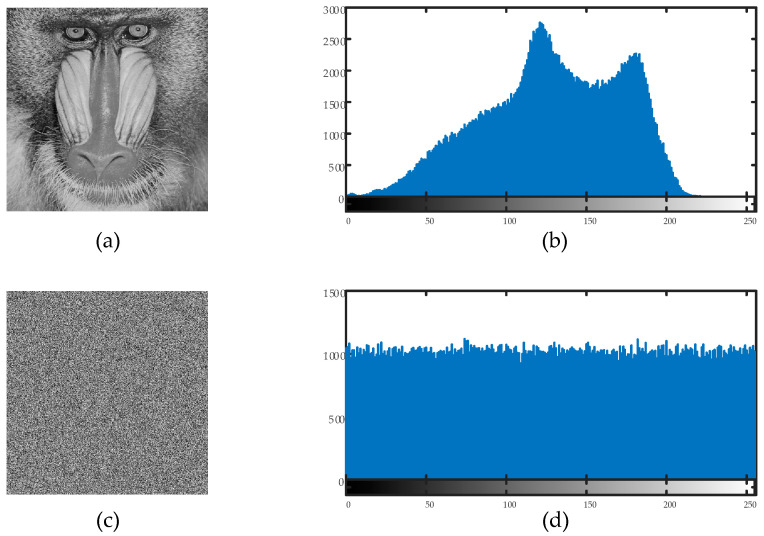
(**a**) Plain baboon image, (**b**) plain baboon image’s histogram; (**c**) encrypted baboon image, (**d**) encrypted baboon image’s histogram.

**Figure 12:**
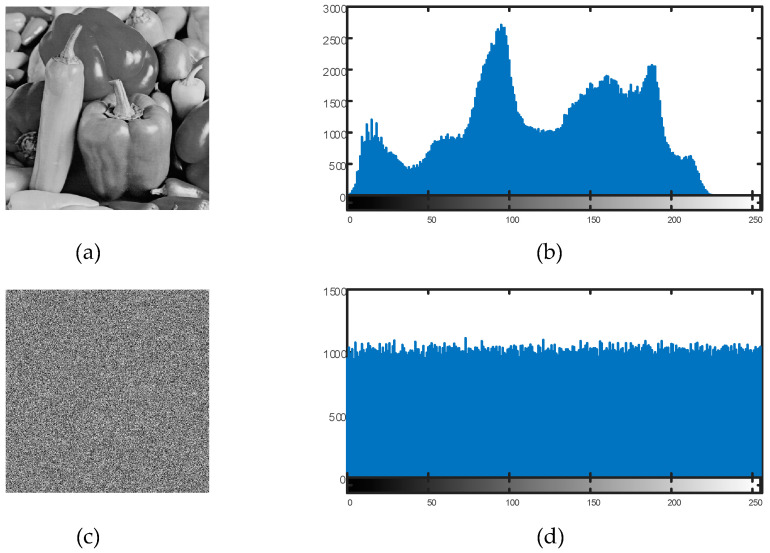
(**a**) Plain pepper image, (**b**) plain pepper image’s histogram; (**c**) encrypted pepper image, (**d**) encrypted pepper image’s histogram.

Histogram studies of the plain and encrypted images were carried out in order to confirm the efficacy of the suggested encryption technique. The distribution of the pixel values in an image is represented by a histogram. To ensure resistance against statistical attacks, an efficient image encryption technique necessitates that the encrypted image’s histogram be uniformly distributed. Figure 11 and Figure 12 display the findings of the histogram analysis.

The encrypted image’s pixel values were uniformly distributed throughout the 0–255 range, as can be plainly seen. This demonstrates that the suggested encryption technique successfully removes discernible histogram trends, strengthening the defenses against statistical attacks.

### 6.3. Information Entropy

Information entropy, which represents the randomness of pixel intensity values, has a theoretical value of 8 for an encrypted image. Uncertainty in encrypted images is assessed using information entropy, a measure of unpredictability. Equation (8) provides the formula for calculating information entropy [60].

(8) $$H\left(m\right)=-\sum_{i=1}^{n}P\left(i\right)\log_{2}P(i)$$

The encrypted image’s information entropy values were 7.9993 for the baboon image and 7.9994 for the pepper image, both of which were very close to the optimal value of 8. These results indicate that the proposed encryption algorithm successfully generates a high degree of randomness, ensuring a robust encryption process.

### 6.4. Differential Attack Resistance

The number of pixel change rate (NPCR) and unified average changing intensity (UACI) measures are used to assess how minor modifications to the plaintext affect the cipher text [61]. As the NPCR value becomes closer to 99.61%, which is regarded as the ideal threshold, the encryption scheme’s sensitivity to plaintext changes increases. In a similar vein, the encryption algorithm’s resistance to differential attacks increases with the UACI value’s proximity to 33.46%. The NPCR and UACI formulas are provided in (9) and (10), respectively.

(9) $$NPCR=\frac{\sum_{i,j}int\left(C\left(i,j\right)\neq C^{\prime }\left(i,j\right)\right)}{M\times N}\times 100\%$$

(10) $$UACI=\frac{1}{M\times N}\left[\sum_{i,j}\frac{\left|C\left(i,j\right)-C^{\prime }\left(i,j\right)\right|}{255}\right]\times 100\%$$

One pixel value in the original image was changed throughout the computation process, and the encrypted version of the original image that had not been altered was compared to the encrypted version of the modified image. For the baboon image, NPCR and UACI were calculated to be 99.6143% and 33.4691%, respectively. Similarly, for the pepper image, the NPCR and UACI values were 99.6143% and 33.3766%, respectively. These results indicate that the proposed encryption algorithm demonstrates strong resistance against differential attacks, as the obtained values were very close to the optimal thresholds.

### 6.5. Correlation Analysis

Only when there is little association between adjacent pixels in the encrypted image can encryption techniques resist statistical analysis attacks. The correlation strength of an image decreases as the correlation coefficient becomes lower. In other words, for an encryption scheme to be resistant to statistical attacks, the correlation coefficient should be close to zero [62]. The correlation coefficient for images is calculated using the set of formulas provided in (11).

(11) $$E\left(x\right)=\frac{1}{N}\sum_{i=1}^{N}x_{i}\;D\left(x\right)=\frac{1}{N}\sum_{i=1}^{N}{\left(x_{i}-E\left(x\right)\right)}^{2}\;cov\left(x,y\right)=\frac{1}{N}\left(\sum_{i=1}^{N}\left(x_{i}-E\left(x\right))(y_{i}-E\left(y\right)\right)\right)\;r_{x,y}=\frac{cov\left(x,y\right)}{\sqrt{D\left(x\right)}\sqrt{D(y)}}$$

**Figure 13:**
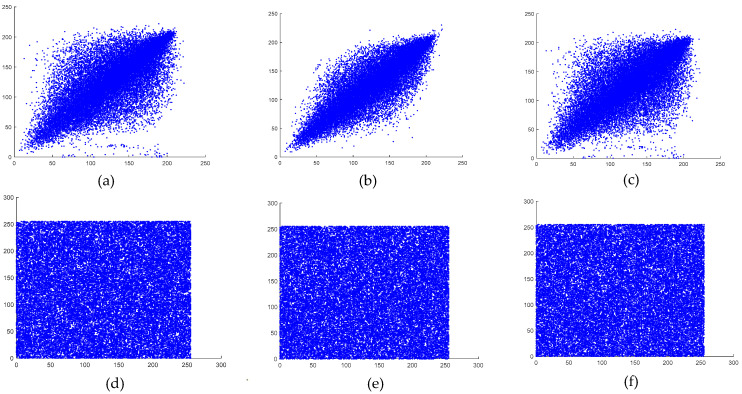
(**a**) Baboon image correlation in plaintext, (**b**) baboon image correlation in plaintext, (**c**) baboon image correlation in plaintext, (**d**) encrypted baboon image correlation in the horizontal direction, (**e**) encrypted baboon image correlation in the vertical direction, and (**f**) encrypted baboon image correlation in the diagonal direction.

**Figure 14:**
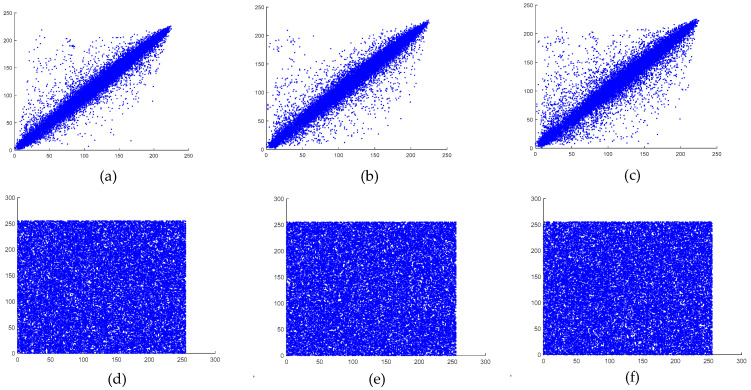
(**a**) Pepper image correlation in plaintext, (**b**) pepper image correlation in plaintext, (**c**) pepper image correlation in plaintext, (**d**) encrypted pepper image correlation in the horizontal direction, (**e**) encrypted pepper image correlation in the vertical direction, and (**f**) encrypted pepper image correlation in the diagonal direction.

Here, x and y represent two consecutive pixel values, while N denotes the number of selected pixel pairs. The vertical, horizontal, and diagonal correlations of both the original and the encrypted images are illustrated in Figure 13 and Figure 14.

As observed, the encrypted images exhibited weak correlations in all directions, whereas the plaintext images maintained strong correlations across all orientations. The calculated correlation coefficients for the plaintext and encrypted images of both the baboon and the pepper images are presented in Table 9.

### 6.6. Keyspace and Key Sensitivity

The proposed encryption algorithm utilizes two parameters and four initial conditions as secret keys, resulting in a highly secure cryptographic system. Assuming that all values use double-precision data, the total key space of the cryptosystem is approximately $10^{96}\approx 2^{318}$. This extensive key space significantly exceeds the commonly accepted security benchmark of $2^{100}$, demonstrating the proposed algorithm’s strong resistance against brute-force attacks. The wide key space ensures that the probability of an unauthorized party guessing the correct key is extremely low, enhancing the overall security and robustness of the encryption scheme.

**Figure 15:**
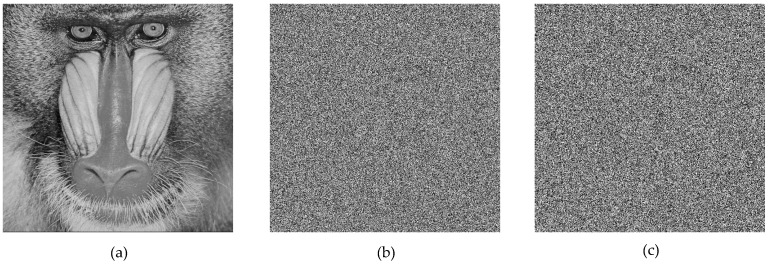
(**a**) Original baboon image, (**b**) encrypted baboon image, (**c**) decrypted baboon image using a separate key.

**Figure 16:**
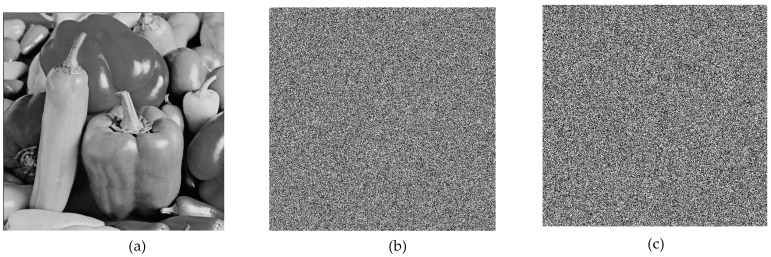
(**a**) Original pepper image, (**b**) encrypted pepper image, (**c**) decrypted pepper image using a separate key.

A strong encryption method needs to have high key sensitivity, which means that even a small alteration to the encryption key should produce a completely different decryption output, making it impossible to successfully decrypt the encrypted image. The decryption process under altered key conditions is illustrated in Figure 15 and Figure 16.

As seen in Figure 15 and Figure 16, even the slightest modification in the decryption key caused significant errors in the recovered images. This confirmed that the proposed encryption scheme exhibits strong key sensitivity.

### 6.7. Plaintext Attacks

**Figure 17:**
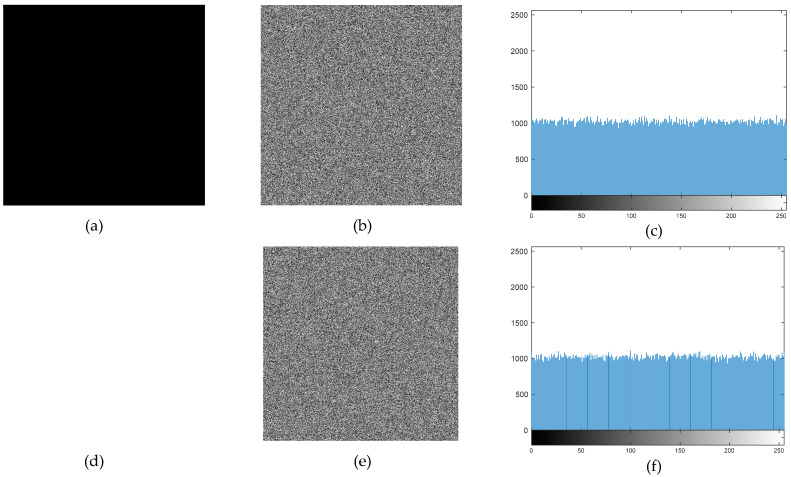
(**a**) Pure black image, (**b**) pure black encrypted image, (**c**) pure black encrypted image histogram, (**d**) pure white image, (**e**) pure white encrypted image, (**f**) pure white encrypted image histogram.

To examine the encryption result, an attacker can try to encrypt a distinct plaintext. Information pertaining to keys may be extracted by the attacker if the encryption technique has flaws. Consequently, even when confronted with a completely black or white plaintext image, the encryption output should continue to be extremely safe. The results of the plaintext attack tests are presented in Figure 17.

The encryption results of pure black and white images confirmed that no anomalies were present, ensuring that the algorithm does not exhibit pattern weaknesses. Additionally, the histogram analysis demonstrated a highly uniform pixel distribution in the encrypted images, confirming resistance against statistical analysis.

### 6.8. Robustness

**Figure 18:**
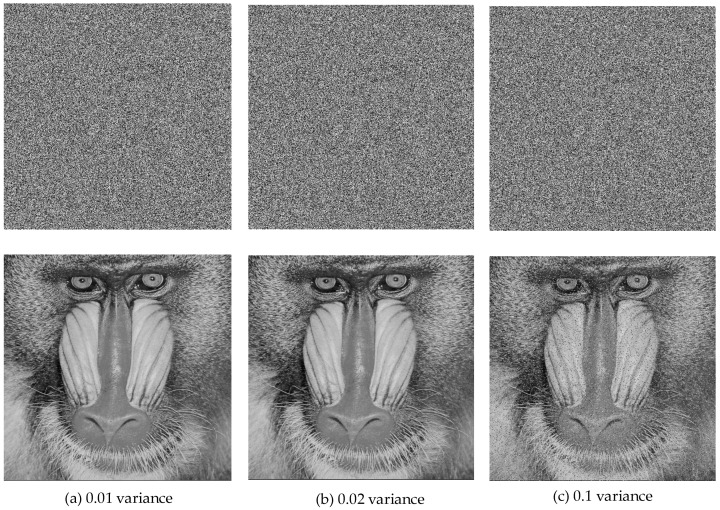
Gaussian noise attack test results.

In real-time encryption applications, encrypted images may experience data loss or noise interference during transmission. A strong encryption method should possess error tolerance and resilience to attacks, ensuring that even if portions of the encrypted data are lost, partial image information can still be recovered during the decoding process. The performance analyses to be performed at this stage were performed using only the 512 × 512 grayscale baboon image. Figure 18 displays the outcomes of the Gaussian noise attack on the decrypted images.

**Figure 19:**
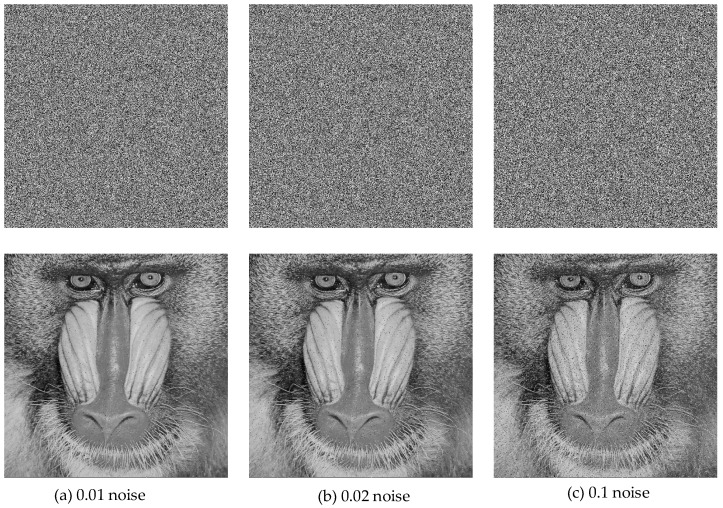
Salt-and-pepper noise attack test results.

As seen in Figure 18, the proposed decryption method performs well under Gaussian noise interference. Similarly, Figure 19 displays the results of the salt-and-pepper noise attack test.

The decryption process under the salt-and-pepper noise attack also yielded satisfactory results. The decrypted image retained all the essential details of the original image, demonstrating exceptional robustness of the proposed encryption technique.

**Figure 20:**
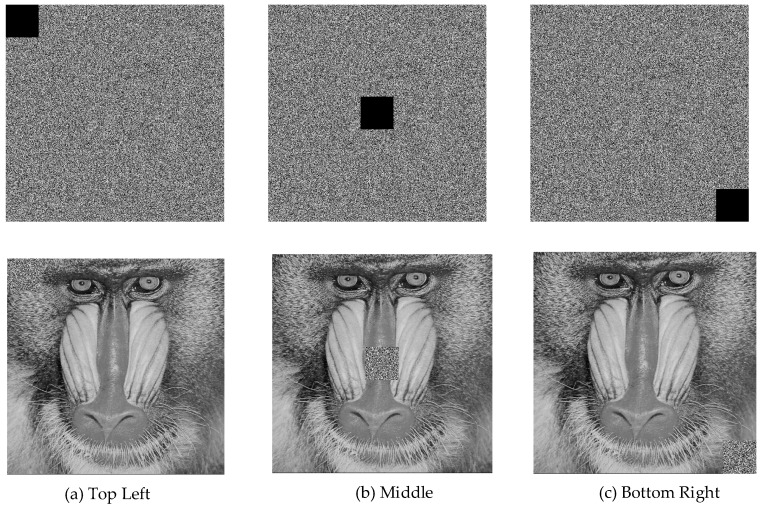
Decryption under cipher text loss in different areas.

The decryption scenario under cipher text loss is illustrated in Figure 20.

It was observed that the proposed encryption scheme successfully resisted image cropping attacks. The decryption process remained unaffected in areas outside the data loss region, while only the cropped section exhibited decoding errors. This confirmed that the proposed approach provides partial resistance against data loss attacks, as it accurately recovers other regions while limiting distortion to the affected area.

### 6.9. Comparison with Other Studies

This section compares the suggested encryption method’s test results with those of previous research. The security evaluation comparison with other works, including both the baboon and the pepper images, is presented in Table 10.

When comparing the proposed method with other studies, recent research conducted in recent years was taken into consideration. The comparative results showed that the suggested encryption strategy outperforms earlier studies in terms of security and efficiency, achieving more successful performance metrics when compared to other modern encryption techniques.

## 7. Conclusions

In this research, the dynamic analysis results of a hyperchaotic system, previously reported in the literature [42], were presented. Instead of reconfirming the hyperchaotic behavior of the system, this study focused on demonstrating its practical application in cryptography. Using this system, a PRNG was designed, and NIST SP800-22 tests were performed. The test results demonstrated that the generated pseudo-random number sequence was statistically reliable and successfully passed the required tests.

A 16×16 S-box was also constructed using the same hyperchaotic system, and its performance was analyzed based on multiple cryptographic criteria. When the results were contrasted with those of other S-box designs, it was discovered that the suggested S-box performed better.

Using the proposed PRNG and S-box, a new image encryption algorithm was developed. The encryption algorithm’s performance was evaluated by applying it to 512×512 grayscale images, including the commonly used baboon and pepper images. After the encryption and decryption processes, the original images were successfully reconstructed. The study conducted detailed security and performance analyses of the encryption algorithm, comparing it with existing methods in the literature. The findings show that, in comparison to existing methods, the suggested image encryption algorithm offers better performance and increased security.

## Figures and Tables

**Table 1:** Results of the NIST SP800-22 test for PRNG.

No	Test	*p*-Value	State
1	Frequency	0.79332	☑
2	Block frequency	0.79374	☑
3	Cumulative sums	0.93912	☑
4	Runs	0.24684	☑
5	Longest run of ones	0.88293	☑
6	Rank	0.13927	☑
7	DFT	0.25135	☑
8	Non-overlapping template	0.56481	☑
9	Overlapping template	0.58974	☑
10	Universal statistical	0.26461	☑
11	Approximate entropy	0.42699	☑
12	Random excursion	0.94021	☑
13	Random excursion variant	0.96653	☑
14	Serial	0.49535	☑
15	Linear complexity	0.30369	☑

**Table 2:** The generated 16×16 S-box.

226	246	176	36	217	100	199	84	157	231	114	91	166	51	223	204
25	57	37	146	14	8	39	49	24	75	186	73	201	89	249	107
170	135	215	238	117	252	178	152	3	18	230	185	173	26	112	30
111	23	98	130	154	118	247	233	216	95	56	59	194	124	219	33
43	142	183	7	81	254	77	31	129	34	133	45	74	147	228	96
50	10	184	189	245	15	192	110	2	5	128	229	222	171	168	127
104	119	188	126	244	62	70	21	169	255	144	149	35	227	150	196
93	234	134	158	243	190	85	87	225	92	52	28	122	44	172	86
200	253	13	22	162	209	241	136	248	106	121	46	211	27	123	0
64	83	82	203	102	214	99	4	160	132	141	180	148	68	139	6
40	16	97	191	153	205	220	202	197	151	55	113	58	105	187	12
155	108	67	143	38	165	120	88	232	174	221	41	60	19	235	175
71	54	1	109	182	240	32	206	11	76	20	65	61	224	90	207
159	239	116	177	181	63	210	125	236	140	145	195	53	138	101	237
163	208	78	213	137	69	80	66	250	79	218	29	72	167	47	198
131	179	212	9	48	17	103	164	42	156	242	193	94	251	115	161

**Table 3:** Analysis of nonlinearity in the S-box proposal.

Methods	1	2	3	4	5	6	7	8	Average Nonlinearity
Proposed S-Box	102	104	106	106	108	108	106	104	105.50

**Table 4:** Independence matrix of the proposed S-box.

0.4531	0.5000	0.5156	0.4844	0.4688	0.4688	0.4844	0.5312
0.4688	0.3906	0.4688	0.5000	0.4688	0.5156	0.5156	0.4062
0.4375	0.5469	0.5000	0.5938	0.4531	0.5312	0.5000	0.5312
0.5469	0.5312	0.5156	0.4844	0.5312	0.5312	0.5469	0.5000
0.5156	0.5312	0.5000	0.3906	0.5781	0.4688	0.4688	0.4844
0.5781	0.4844	0.5469	0.4688	0.5000	0.5000	0.5312	0.4219
0.4844	0.5469	0.4844	0.4688	0.5469	0.5469	0.4531	0.5312
0.4531	0.5312	0.4219	0.4844	0.5312	0.4688	0.4844	0.5469

**Table 5:** Values of the proposed S-box in BIC-NL.

0	100	104	98	104	104	108	96
100	0	102	102	102	104	102	104
104	102	0	106	104	104	104	102
98	102	106	0	102	104	108	104
104	102	104	102	0	104	104	104
104	104	104	104	104	0	106	102
108	102	104	108	104	106	0	104
96	104	102	104	104	102	104	0

**Table 6:** Values of the suggested S-box’s BIC-SAC.

0.0	0.5312	0.4219	0.4844	0.5312	0.4688	0.4844	0.5469
0.4844	0.0	0.4844	0.4688	0.5469	0.5469	0.4531	0.5312
0.5781	0.4844	0.0	0.4688	0.5000	0.5000	0.5312	0.4219
0.5156	0.5312	0.5000	0.0	0.5781	0.4688	0.4688	0.4844
0.5469	0.5312	0.5156	0.4844	0.0	0.5312	0.5469	0.5000
0.4375	0.5469	0.5000	0.5938	0.4531	0.0	0.5000	0.5312
0.4688	0.3906	0.4688	0.5000	0.4688	0.5156	0.0	0.4062
0.4531	0.5000	0.5156	0.4844	0.4688	0.4688	0.4844	0.0

**Table 7:** The suggested S-box’s DP table.

6	6	8	6	8	8	6	8	6	6	6	6	8	6	10	6
8	6	6	6	8	8	6	6	6	6	6	8	8	6	6	6
6	10	6	6	6	6	6	8	6	8	6	6	6	12	6	6
8	8	6	6	6	8	6	6	6	8	6	6	6	6	10	8
6	8	8	6	6	8	6	8	6	8	10	8	6	6	8	6
6	8	8	8	6	6	6	6	6	6	6	6	6	6	8	6
8	6	6	6	8	8	8	6	6	6	6	6	6	8	8	6
6	8	8	6	8	8	6	8	6	6	8	10	6	8	8	8
8	6	8	6	10	6	6	6	8	10	6	6	10	8	8	8
6	6	6	6	6	8	10	8	8	6	6	8	8	8	8	6
6	8	6	10	8	6	8	6	6	6	8	8	8	6	6	6
6	8	6	8	6	6	8	8	6	6	10	6	6	8	8	6
6	8	6	6	8	6	6	8	6	8	6	6	6	6	6	6
8	6	8	6	8	6	12	6	6	6	8	6	8	6	6	6
8	8	6	6	10	6	6	6	6	8	6	6	6	6	6	8
6	6	8	8	8	10	6	10	8	6	8	6	8	6	6	8

**Table 8:** The proposed S-box and current S-box designs are compared.

S-Box	Nonlinearity			SAC			BIC-SAC	BIC-NL	LP	DP
	Min.	Max.	Avg.	Min.	Max.	Avg.				
Ref. [51]	102.0	108.0	104.30	0.4219	0.5625	0.4923	0.5000	102.70	N/A	10
Ref. [52]	100.0	106.0	104.00	0.4746	0.5390	0.5033	0.4947	103.21	0.125	12
Ref. [53]	102.0	108.0	104.50	0.4219	0.6406	0.4980	0.5075	104.64	0.125	12
Ref. [54]	100.0	108.0	104.50	0.4218	0.6250	0.4978	0.4974	103.64	0.132	12
Ref. [55]	104.0	108.0	105.25	0.3593	0.5937	0.4988	0.5039	102.72	0.132	10
Ref. [56]	98.0	106.0	103.7	0.3910	0.5931	0.4962	0.4623	103.80	0.125	12
Ref. [57]	102.0	108.0	105.25	0.4688	0.5938	0.5003	0.5000	103.21	N/A	14
Ref. [58]	101.0	107.0	104.50	0.4220	0.5780	0.4960	0.4940	103.30	0.140	10
Ref. [59]	100.0	106.0	103.00	0.3900	0.5930	0.5020	0.4990	102.90	0.140	10
Proposed S-Box	102.0	108.0	105.50	0.3906	0.5781	0.4980	0.4976	103.29	0.140	12

**Table 9:** Correlation coefficients of the plaintext and encrypted images.

Image	Plain Image			Encrypted Image		
	Horizontal	Vertical	Diagonal	Horizontal	Vertical	Diagonal
Baboon (512 × 512)	0.7584	0.8688	0.7264	0.0013	0.0016	0.0036
Peppers (512 × 512)	0.9765	0.9782	0.9633	−0.0143	−0.0049	−0.0112

**Table 10:** Comparison with other studies.

Image Encrypted	Differential Attack		Correlation analysis			Information Entropy
	UACI	NPCR	Horizontal	Vertical	Diagonal	
Ref. [63]	33.4949%	99.6246%	0.0007	0.0007	0.0080	7.9974
Ref. [64]	33.4146%	99.6551%	−0.0027	−0.0027	0.0088	7.9974
Ref. [65]	33.6344%	99.6292%	−0.029	0.01009	−0.0099	7.9992
Ref. [66]	33.4997%	99.5972%	0.0019	0.0011	0.0005	7.9993
Ref. [40]	33.4695%	99.6093%	0.0025	0.0015	0.0015	7.9994
Ref. [67]	33.4742%	99.6075%	0.0251	0.0040	0.0231	7.9993
Ref. [68]	33.4039%	99.6016%	−0.0143	0.0112	0.0013	7.9993
512 × 512 Baboon	33.4691%	99.6143%	0.0013	0.0016	0.0036	7.9993
512 × 512 Peppers	33.3766%	99.6143%	−0.0143	−0.0049	−0.0112	7.9994

## Data Availability

The original contributions presented in the study are included in the article, further inquiries can be directed to the corresponding author.
